# Two-year topographic and densitometric outcomes of accelerated (45 mW/cm^2^) transepithelial corneal cross-linking for keratoconus: a case-control study

**DOI:** 10.1186/s12886-018-0999-4

**Published:** 2018-12-27

**Authors:** Jinrong Huang, Yang Shen, Weijun Jian, Haipeng Xu, Meiyan Li, Jing Zhao, Xingtao Zhou, Hongfei Liao

**Affiliations:** 1 The Eye and ENT Hospital of Fudan University, 19 Baoqing Road, Xuhui District, Shanghai, 200031 China; 2 The Key Lab of Myopia, National Health and Family Planning Commission of the People’s Republic of China, Shanghai, China; 3 Department of Ophthalmology, The First Affiliated Hospital of Jiangxi Medical College, Shang Rao, Jiangxi Province China; 4 Department of Ophthalmology, The Affiliated Eye Hospital of Nanchang University, 463 Bayi Avenue, Nanchang, 330006 Jiangxi Province China

**Keywords:** Keratoconus, Accelerated transepithelial corneal cross-linking, Corneal topographic parameters, Densitometry

## Abstract

**Background:**

Conventional corneal cross-linking is effective for retarding the progression of keratoconus. However the long-term efficacy and safety of accelerated (45 mW/cm2) transepithelial corneal cross-linking (ATE-CXL) on progressive keratoconus (KC) treatment is not fully understood. The purpose of this study is to evaluate the 2-year changes in corneal topographic parameters and densitometry values after ATE-CXL for KC.

**Methods:**

Twenty-five progressive eyes of 25 KC patients (KC group) and 25 eyes of 25 myopes without KC (control group) were enrolled. Corneal topography and densitometry values were evaluated pre-operatively and at 6, 12 and 24 months post-operatively in the KC group.

**Results:**

The mean values of flat keratometry (K1), steep keratometry (K2), mean keratometry (Km), corneal astigmatism (CA), maximum keratometry (Kmax), central corneal thickness (CCT), thinnest corneal thickness (TCT), anterior corneal elevation (ACE) and posterior corneal elevation (PCE) all remained unchanged over time (all *P* values > 0.05). The densitometry values of the anterior, central, posterior and total layers over the annular diameters 0 mm to 2 mm (Φ0-2 mm) and Φ2–6 mm all decreased significantly (all *P* values < 0.05). At post-operative month 24, except for the densitometry value of the posterior layer (Φ0-2 mm), which was significantly lower than that of the control group (post hoc *P* = 0.010), all densitometry values obtained from the remaining locations of the KC eyes were equal to those of the control group (All post hoc *P* values > 0.05). Subgroups with Km ≥ 50.30D or ACE ≥35.3 μm progressed significantly when compared with those with Km < 50.30D (F = 8.167, *P* = 0.004) or ACE< 35.3 μm (F = 5.207, *P* = 0.022).

**Conclusions:**

K1, K2, Km, CA, Kmax, CCT, TCT, ACE, and PCE values may remain stable but severer KC patients tend to have poorer long-term outcomes. The densitometry values of the full corneal thickness (total layer over Φ0-2 mm and Φ2–6 mm) may decrease to normal levels at 2 years after ATE-CXL for KC.

## Introduction

Keratoconus (KC) is a chronic ectatic corneal disease characterized by progressive corneal thinning and steeping which lead to irregular astigmatism and visual impairment [1]. Treatment options, including spectacles, rigid gas permeable contact lenses (RGP) [2], intrastromal corneal rings (ICR) [3], keratoplasty [4, 5] and riboflavin ultraviolet-A (UV-A) corneal cross-linking (CXL) [6], are available for improving visual acuity or (and) retarding the progression of KC. Spectacles and RGP correct refractive error but do not change the course of the disease. CXL is effective for slowing or stopping the progression of KC [7]. Literatures have reported that CXL results in increased collagen fiber diameter [8] and enhances biomechanical stiffness [9] through the induction of increased formation of covalent bonds within or between collagen fibers in the corneal stroma [10]. Greenstein SA [11] reported a significant increase in corneal densitometry after conventional (3 mW/cm^2^) corneal crosslinking (C-CXL), and transient sub-epithelial haze is frequently detected after C-CXL [12, 13]. Accelerated (45 mW/cm^2^) trans-epithelial corneal cross-linking with pulsed UV (ATE-CXL) is a novel and minimally invasive corneal cross-linking procedure. Higher irradiance, pulsed UV light is used to dramatically shorten treatment duration as compared with the C-CXL (3 mW/cm^2^) procedure. As the epithelium layer remains intact during ATE-CXL, risk of infection is minimized, and milder ocular surface inflammatory reactions may be triggered, and hence, post-operative complications may be reduced [14]. One of our previous studies [15] investigated one-year outcomes of corneal topographic parameters and corneal densitometry after ATE-CXL for KC. Our results demonstrated decreased corneal densitometry and stable keratometry values at post-operative year 1 [15]. Corneal densitometry, which is also known as corneal backscattering, is considered as a proxy for corneal transparency [16]. It is important to determine whether CXL would lead to loss of transparency when assess the safety of CXL protocols. However the long-term effects of ATE-CXL on corneal densitometry and keratometry remain unknown. In the current study, we investigated 2-year changes in corneal topographic parameters and densitometry values after ATE-CXL for KC.

## Methods

### Ethics statement

This prospective, non-randomized and cohort study was approved by the Ethics Committee of the Eye and ENT Hospital of Fudan University (ethical approval number: ky2012–017. Date of approved by ethic committee: 2012-08-16) and was carried out following the tenets of the Declaration of Helsinki. After a detailed explanation of the study, written informed consent for the treatment was obtained from the patients or their legal representatives before the procedure.

### Patients

Twenty-five progressive KC patients (age 25.4 ± 6.0 years, 18 male and 7 female, Amsler-Krumeich classification stages 1–4, without stromal scarring) and 25 myopes matched for gender and age (within ±2 years) were enrolled in this study. The worse eye (the eye with more advanced keratoconus) of each KC patient underwent ATE-CXL and was followed up for 2 years.

### Ophthalmologic examinations

For KC patients, manifest refraction spherical equivalent (MRSE), best spectacles corrected distance visual acuity (BSCDVA), slit-lamp anterior segment and fundus examinations, and endothelial cell density (SP-2000P; Topcon Corporation, Japan) were assessed pre-operatively. A 3-dimensional anterior segment analyzer (Pentacam HR, Type 70,900; Oculus, Germany) was applied to evaluate the corneal topographic parameters, including flat keratometry value (K1), steep keratometry value (K2), mean keratometry value (Km), corneal astigmatism (CA), maximum keratometry value (Kmax), central corneal thickness (CCT), thinnest corneal thickness (TCT), anterior corneal elevation (ACE) and posterior corneal elevation (PCE), as well as corneal densitometry values, pre-operatively and at 6, 12 and 24 months post-operatively. All corneal densitometry values and corneal topographic parameters were only measured once in the control group. Exclusion criteria of ATE-CXL included TCT of less than 340 μm, endothelial cell density of less than 2000 cells per mm^2^, severe dry eye, corneal scarring, autoimmune diseases or pregnancy [12, 17, 18].

### Surgical techniques

ATE-CXL was performed by one surgeon (XTZ) under topical anesthesia with 3 drops of 0.4% oxybuprocaine hydrochloride (Santen Pharmaceutical Co, Ltd., Osaka, Japan). A lid speculum was applied to expose the whole cornea, and a trephine with an inner diameter of 8.5 mm (Model 52503B; 66 vision Tech Co, Ltd., Suzhou, China) was placed on the cornea. ParaCel Solution (riboflavin 0.25%, hydroxyl-propyl-methyl-cellulose, NaCl, ethylene-diamine-tetra-acetic acid, Tris, and benzalkonium chloride; Medio-Haus-Medizinprodukte GmbH, Kiel, Germany) was instilled into the trephine for 4 min and was removed immediately with a cellulose sponge. Vibex-Xtra Solution (riboflavin phosphate 2.80 mg/mL and NaCl, Avedro, Inc.) was subsequently dripped into the trephine, and the soaking time was set to 6 min. After soaking, the trephine was removed. Then, a 365-nm UV-A light (Avedro’s KXL System; Avedro, Inc) with irradiation intensity of 45 mW/cm^2^ was applied to irradiate the cornea in pulsed mode (1 s on, 1 s off) for 5 min and 20 s, for a total energy dose of 7.2 J/cm^2^. Balanced salt solution was administered every 40 s for ocular surface protection during the irradiation. A bandage contact lens (Acuvue Oasys, Inc., Jacksonville, FL) was placed on the cornea after the procedure and was left in place for 3 days.

### Post-operative topical medication regimens

Each patient was instructed to instill topical antibiotics (ofloxacin ophthalmic solution 0.5%; Santen Pharmaceutical Co, Ltd.; 4 times per day for 7 days), artificial tears (TearsNaturale; Alcon Laboratories, Inc., Fort Worth, TX; 4 times per day for 20 days) and topical steroids (fluorometholone 0.1%; Santen Pharmaceutical Co, Ltd.; 8 times per day initially, with gradually reduced frequencies for a period of 20 days), post-operatively.

### Statistical evaluation

Statistical analysis was performed using SPSS 20 software (SPSS Inc., IBM). Kolmogorov-Smirnov test was conducted to assess the normality of these variables. Mixed linear model with Bonferroni-adjusted post hoc comparisons were performed to evaluate the changes in corneal topographic parameters and corneal densitometry over time, as well as the difference in corneal densitometry between groups. The potential correlations between the corneal topographic parameters and the densitometry values were evaluate by using Pearson correlation test. Cut-off *P* values were 0.05.

## Results

In KC group, all procedures were uneventful (*n* = 25). Two patients failed to attend the follow-up of post-operative month 6 (*n* = 23). At post-operative month 12, 1 patient withdrew from the study, and 1 additional patient did not come for the visit (n = 23). At post-operative month 24, another 2 patients failed to attend the last visit (*n* = 22). The reasons for loss of follow-up included inconvenient traffic and personal affairs (work- or school-related problems). No statistically significant difference was detected in the mean values of age between the two groups (*t* = − 0.440, *P* = 0.664).

### Changes in main corneal topographic parameters after ATE-CXL

The pre-operative and post-operative corneal topographic parameters, including K1, K2, Km, Kmax, CA, ACE, PCE, CCT and TCT are listed in the Table 1. All these variables remained stable over time (All *P* values > 0.05). At post-operative month 24 (*n* = 22), 12 out of 25 (48%) treated eyes had deceased Kmax values, 3 out of 25 (12%) eyes had < 1 diopter (D) increase in the Kmax values, and the remaining 7 (28%) eyes showed progression (≥ 1 diopter (D) increase in the Kmax value within 2 years). During the follow-ups, the mean values of K1, Km and Kmax increased non-statistically from 48.69D to 49.72D (F = 1.556, *P* = 0.227), from 49.90D to 50.78D (F = 1.051, *P* = 0.389) and from 59.12D to 60.76D (F = 0.656, *P* = 0.588), respectively. K2 and CA rose modestly at post-operative month 12 and then fell slightly at post-operative month 24 (F = 0.601, *P* = 0.621 and F = 1.772, *P* = 0.181, respectively). The mean values of CCT and TCT both decreased slightly at post-operative month 12 when compared with the pre-operative values, and then increased modestly at post-operative month 24 (F = 1.167,*P* = 0.344 and F = 0.711, *P* = 0.556, respectively). The mean values of ACE and PCE fluctuated from 31.6 μm to 35.2 μm and from 61.6 μm to 65.5 μm (F = 1.091, *P* = 0.372 and F = 0.025, *P* = 0.994), respectively.

**Table 1:** Changes in Main Corneal Topographic Parameters

Variables	Pre-operation		6 months Post-operation		12 months Post-operation		24 months Post-operation		F^a^	*P*
	(*n* = 25)		(*n* = 23)		(*n* = 23)		(*n* = 22)			
	Mean ± SD	Range	Mean ± SD	Range	Mean ± SD	Range	Mean ± SD	Range		
K1 (D)	48.69 ± 5.15	41.30 to 58.20	49.17 ± 5.68	40.80 to 59.10	49.43 ± 5.59	41.00 to 59.50	49.72 ± 5.60	41.10 to 60.90	1.556	0.227
K2 (D)	51.17 ± 5.50	42.60 to 61.00	51.60 ± 5.64	41.80 to 61.00	52.05 ± 5.64	42.40 to 61.30	51.76 ± 5.21	41.90 to 61.10	0.601	0.621
Km (D)	49.90 ± 5.24	41.90 to 59.00	50.34 ± 5.57	41.30 to 59.50	50.70 ± 5.51	41.70 to 60.30	50.78 ± 5.26	41.50 to 61.00	1.051	0.389
CA (D)	2.48 ± 2.04	0.00 to 7.40	2.35 ± 1.88	0.40 to 7.00	2.62 ± 2.04	0.20 to 7.20	2.17 ± 1.91	0.10 to 6.40	1.772	0.181
Kmax (D)	59.12 ± 8.80	43.30 to 78.00	59.21 ± 9.10	42.80 to 75.70	59.88 ± 9.25	43.40 to 76.30	60.76 ± 8.87	43.20 to 76.00	0.656	0.588
CCT (μm)	466.6 ± 37.6	348.0 to 527.0	462.8 ± 42.6	335.0 to 522.0	461.3 ± 40.6	335.0 to 511.0	465.6 ± 43.8	335.0 to 532.0	0.553	0.652
TCT (μm)	447.7 ± 39.7	320.0 to 510.0	442.7 ± 49.6	305.0 to 515.0	440.2 ± 42.6	305.0 to 501.0	448.9 ± 45.1	305.0 to 512.0	2.021	0.139
ACE (μm)	34.0 ± 18.4	5.0 to 75.0	32.6 ± 17.4	3.0 to 69.0	35.2 ± 20.2	6.0 to 80.0	31.6 ± 14.7	8.00 to 58.00	1.112	0.365
PCE (μm)	62.6 ± 27.4	13.0 to 114.0	61.6 ± 27.6	11.0 to 111.0	65.5 ± 27.1	14.0 to 112.0	63.9 ± 24.7	20.00 to 108.0	0.135	0.938

*D* Diopter, *μm* Micrometer, *K1* Flat keratometry, *K2* Steep keratometry, *Km* Mean keratometry, *CA* Corneal astigmatism, *Kmax* Maximum keratometry, *CCT* Central corneal thickness, *TCT* Thinnest corneal thickness, *ACE* Anterior corneal elevation, *PCE* Posterior corneal elevation, ^a^ Mixed linear model analysis

### Changes in corneal densitometry values after ATE-CXL

**Fig. 1:**
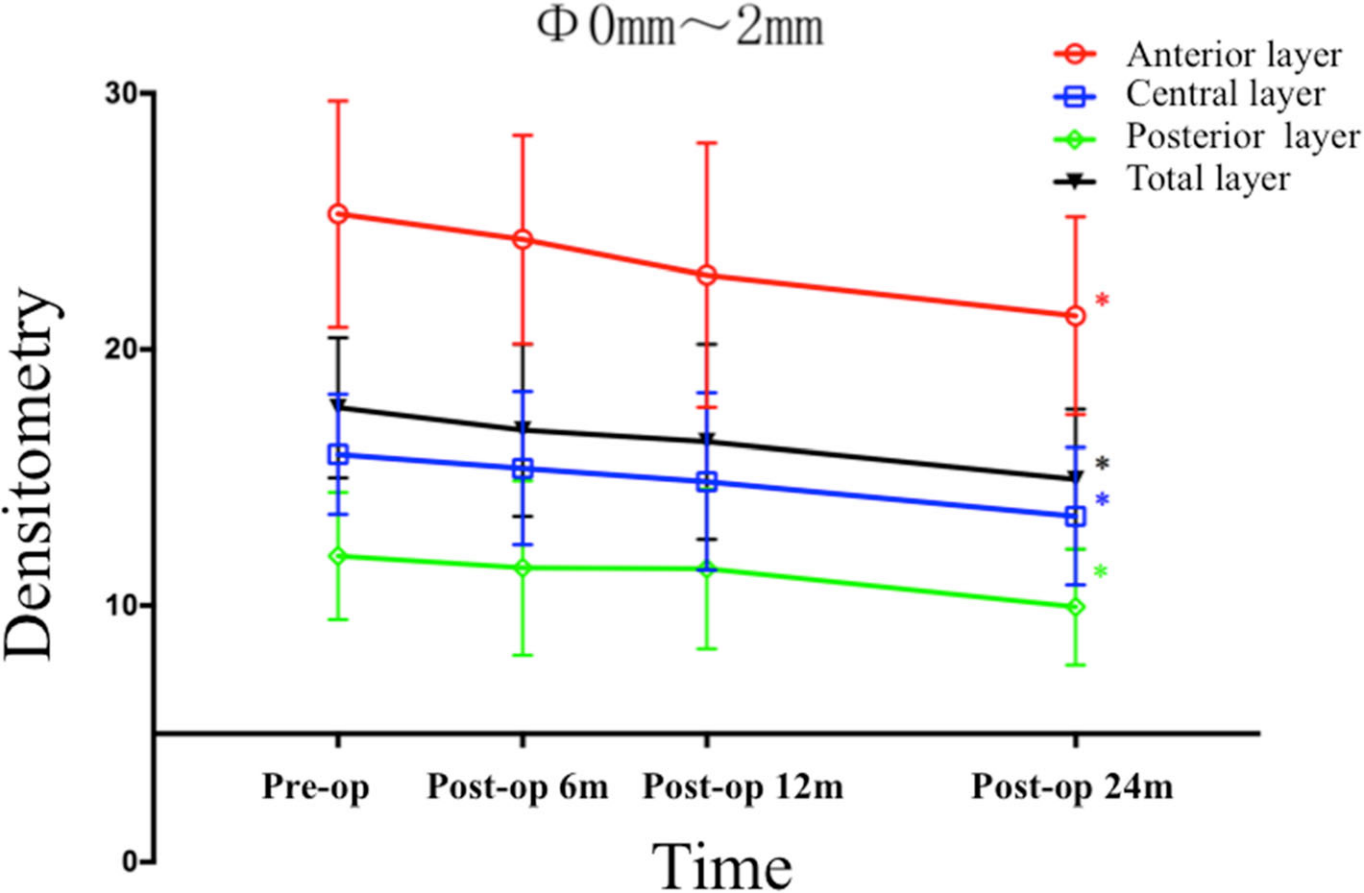
The densitometry values over Φ0-2 mm of the anterior layer, central layer, posterior layer, and total layer of the corneas measured before and at 6, 12 and 24 months after ATE-CXL (The asterisk refers to a significant difference that was detected when compared with the preoperative value)

**Fig. 2:**
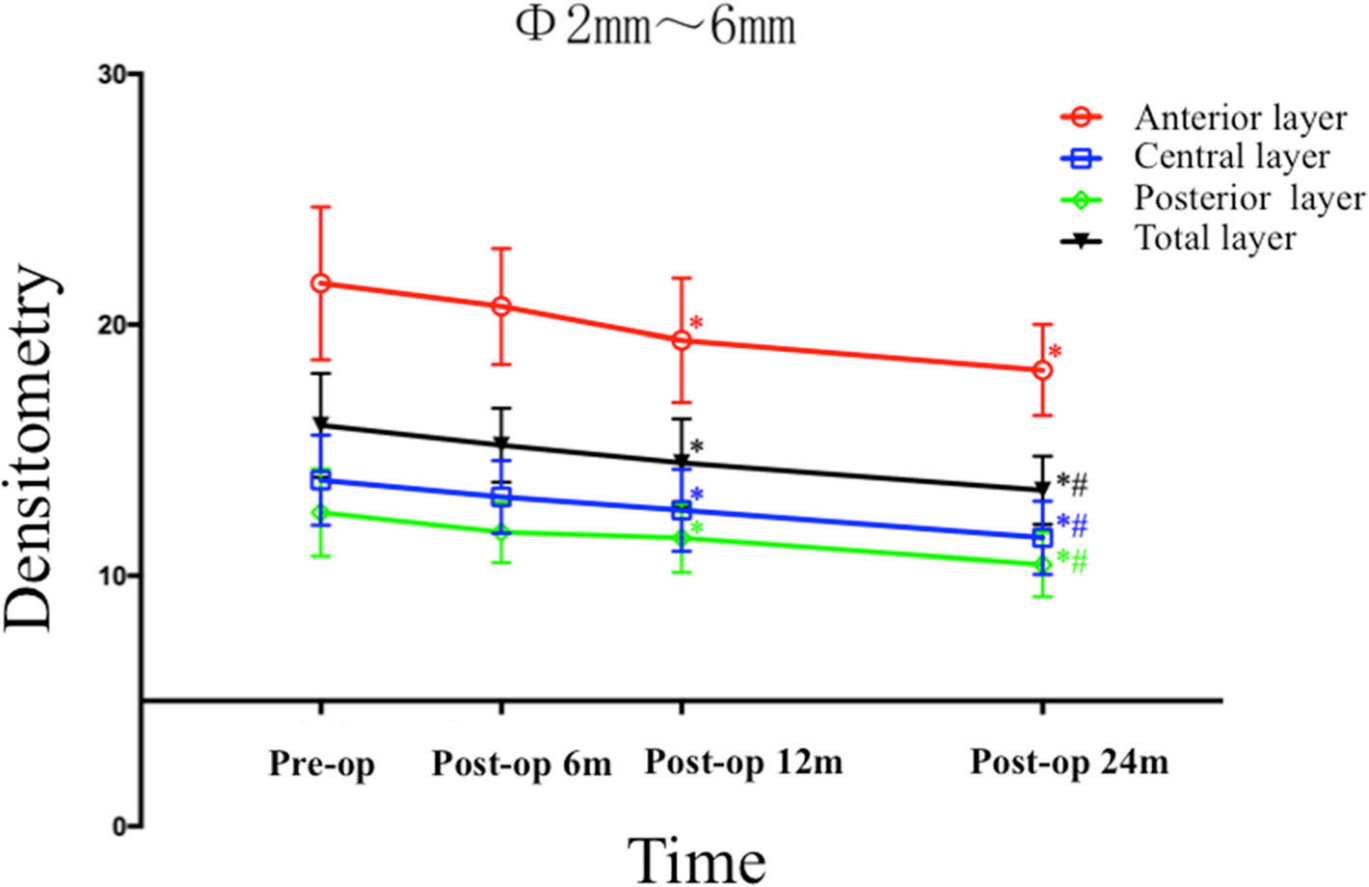
The densitometry values over Φ2–6 mm of the anterior layer, central layer, posterior layer, and total layer of the corneas measured before and at 6, 12 and 24 months after ATE-CXL. (The asterisk refers to a significant difference that was detected when compared with the preoperative value. The octothorpe refers to a significant difference that was detected when compared with the value obtained at post-operative month 12)

As listed in Table 2, the densitometry values of the anterior layer, the central layer, the posterior layer, and the total layer over Φ 0 to 2 mm, as well as over Φ 2 to 6 mm, all decreased significantly over time (All *P* values < 0.001). Bonferroni post hoc comparisons showed densitometry values dramatically decreased in the anterior layer (post hoc *P* = 0.002), the central layer (post hoc P = 0.002), the posterior layer (post hoc *P* = 0.003) and the total layer (post hoc *P* = 0.001) over Φ 0 to 2 mm at post-operative month 24, when compared with the pre-operative values (Fig. 1). As illustrated in Fig. 2, the mean densitometry values of the anterior layer, central layer, posterior layer and total layer over Φ 2 to 6 mm all fell remarkably at post-operative month 12 (post hoc *P* = 0.005, post hoc *P* = 0.003, post hoc P = 0.003 and post hoc P = 0.003, respectively), when compared with the pre-operative values. Except for the densitometry value obtained from the anterior layer over Φ2–6 mm, which changed insignificantly (post hoc *P* = 0.052), the densitometry values obtained from the central layer, the posterior layer and the total layer over Φ2–6 mm all significantly decreased at post-operative month 24 when compared with the values obtained at post-operative month 12 (post hoc *P* = 0.001, post hoc *P* = 0.002 and post hoc *P* = 0.003, respectively).

**Table 2:** Changes in Corneal Densitometry

Locations	Pre-operation		6 months Post-operation		12 months Post-operation		24 months Post-operation		F^a^	*P*
	(*n* = 25)		(*n* = 23)		(*n* = 23)		(*n* = 22)			
	Mean ± SD	Range	Mean ± SD	Range	Mean ± SD	Range	Mean ± SD	Range		
Anterior layer	25.28 ± 4.42	19.90 to 36.10	24.29 ± 4.08	18.70 to 38.10	22.90 ± 5.16	16.7 to 42.7	21.31 ± 3.86	16.90 to 36.20	19.302	< 0.001^b^
(Φ 0–2 mm)										
Anterior layer	21.66 ± 3.05	17.20 to 29.20	20.73 ± 2.31	17.00 to 26.40	19.38 ± 2.48	16.40 to 24.50	18.20 ± 1.82	15.30 to 22.20	34.316	< 0.001^b^
(Φ 2–6 mm)										
Central layer	15.91 ± 2.34	12.00 to 23.00	15.37 ± 2.99	11.00 to 27.40	14.84 ± 3.46	10.80 to 28.30	13.49 ± 2.68	10.70 to 23.50	22.204	< 0.001^b^
(Φ 0–2 mm)										
Central layer	13.80 ± 1.80	10.50 to 18.30	13.13 ± 1.45	10.10 to 16.50	12.60 ± 1.64	9.60 to 15.60	11.51 ± 1.46	9.70 to 15.20	20.295	< 0.001^b^
(Φ 2–6 mm)										
Posterior layer	11.94 ± 2.49	6.00 to 16.20	11.48 ± 3.41	5.80 to 24.40	11.44 ± 3.11	6.60 to 21.40	9.95 ± 2.26	6.50 to 15.50	10.309	< 0.001^b^
(Φ 0–2 mm)										
Posterior layer	12.51 ± 1.74	9.70 to 16.90	11.73 ± 1.21	8.80 to 14.70	11.49 ± 1.36	8.80 to 14.80	10.44 ± 1.29	8.30 to 12.70	15.864	< 0.001^b^
(Φ 2–6 mm)										
Total layer	17.72 ± 2.74	13.30 to 25.10	16.85 ± 3.36	11.80 to 30.00	16.39 ± 3.81	11.30 to 30.80	14.94 ± 2.74	11.70 to 25.10	16.972	< 0.001^b^
(Φ 0–2 mm)										
Total layer	15.99 ± 2.08	12.70 to 21.30	15.20 ± 1.48	12.90 to 19.20	14.49 ± 1.75	11.70 to 18.30	13.40 ± 1.37	11.50 to 16.50	26.231	< 0.001^b^
(Φ 2–6 mm)										

*Φ* Annular diameters, ^a^ Mixed linear model analysis, ^b^A significant difference was detected

**Fig. 3:**
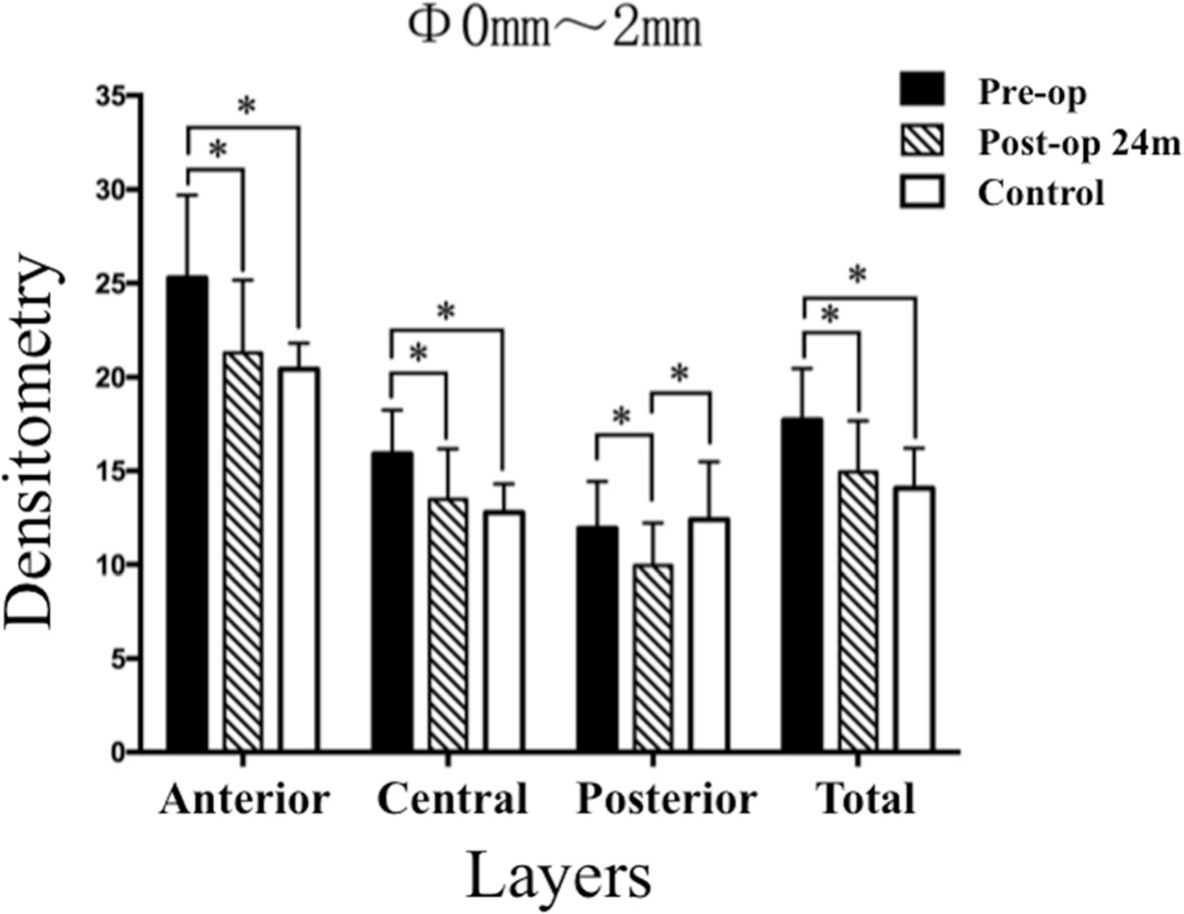
The mean densitometry values over Φ0-2 mm of preoperation, 24-month postoperation, and the control group (The asterisk refers to that a significant difference was detected between the two groups)

**Fig. 4:**
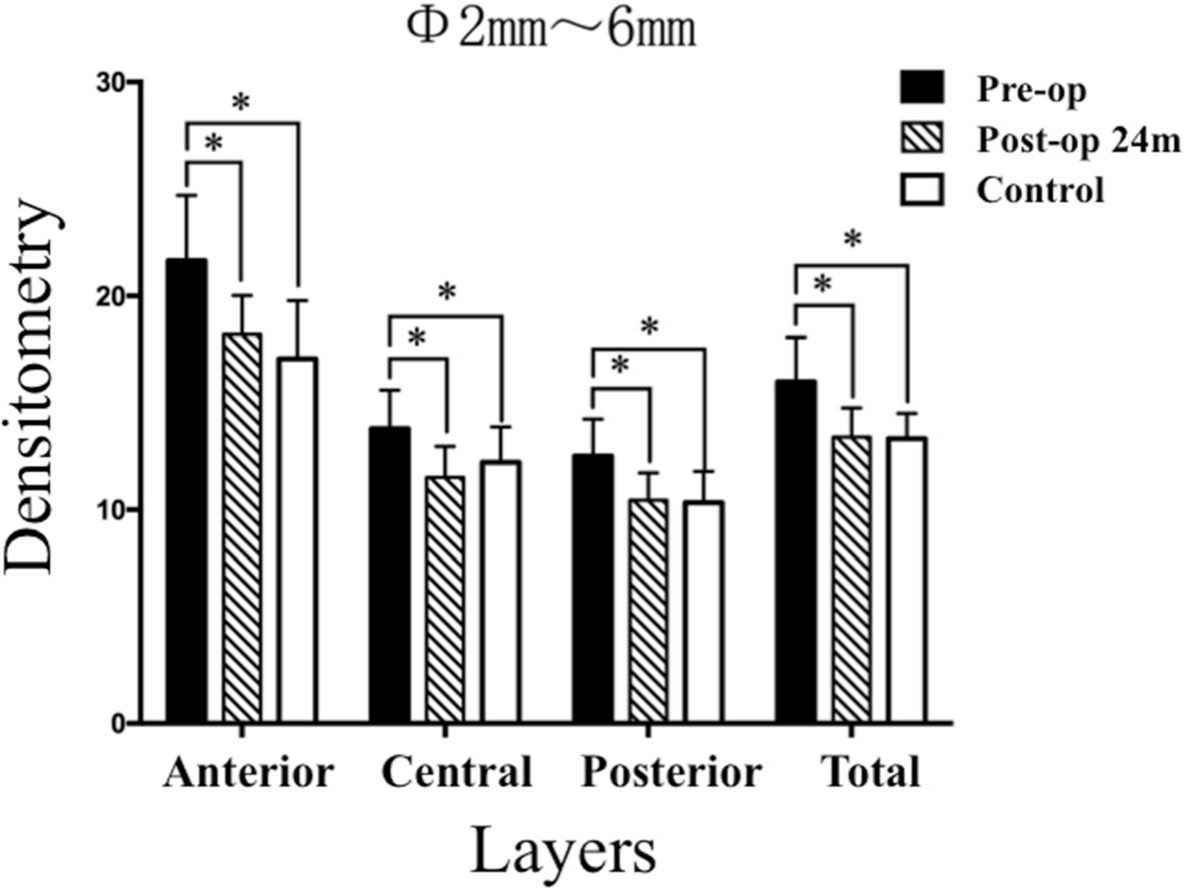
The mean densitometry values over Φ2–6 mm of preoperation, 24-month postoperation, and the control group (The asterisk refers to that a significant difference was detected between the two groups)

As demonstrated in Table 3, mixed linear model analysis revealed significant difference in densitometry values between groups (All *P* values ≤  0.001). Bonferroni-adjusted post hoc comparisons detected that the densitometry values obtained in the anterior layer, the central layer, the posterior layer and the total layer, over both Φ0-2 mm and Φ2–6 mm at post-operative month 24, were significantly decreased when compared with the values obtained pre-operatively (All post hoc *P* values < 0.001). Except for the densitometry value of the posterior layer over Φ0-2 mm, obtained at post-operative month 24, was significantly lower than that of the control group (post hoc *P* = 0.010), the densitometry values of the remaining locations (Figs. 3 and 4) were similar to the values obtained in the control group (All post hoc P values > 0.05).

**Table 3:** Differences in Corneal Densitometry Between Groups

Locations	Pre-operation (*n* = 25)	24 months Post-operation	Control	F^a^	*P*
		(*n* = 22)	(*n* = 25)		
	Mean ± SD	Mean ± SD	Mean ± SD		
Anterior layer (Φ 0–2 mm)	25.28 ± 4.42	21.31 ± 3.86	20.44 ± 1.35	14.608	< 0.001^b^
Anterior layer (Φ 2–6 mm)	21.66 ± 3.05	18.20 ± 1.82	17.07 ± 2.71	22.590	< 0.001^b^
Central layer (Φ 0–2 mm)	15.91 ± 2.34	13.49 ± 2.68	12.78 ± 1.53	17.215	< 0.001^b^
Central layer (Φ 2–6 mm)	13.80 ± 1.80	11.51 ± 1.46	12.22 ± 1.66	20.404	< 0.001^b^
Posterior layer (Φ 0–2 mm)	11.94 ± 2.49	9.95 ± 2.26	12.39 ± 3.09	9.130	=0.001^b^
Posterior layer (Φ 2–6 mm)	12.51 ± 1.74	10.44 ± 1.29	10.33 ± 1.47	21.574	< 0.001^b^
Total layer (Φ 0–2 mm)	17.72 ± 2.74	14.94 ± 2.74	14.07 ± 2.14	15.995	< 0.001^b^
Total layer (Φ 2–6 mm)	15.99 ± 2.08	13.40 ± 1.37	13.33 ± 1.19	22.801	< 0.001^b^

*Φ* Annular diameters, ^a^ Mixed linear model analysis, ^b^A significant difference was detected

### Correlations between topographic parameters and densitometry values

**Fig. 5:**
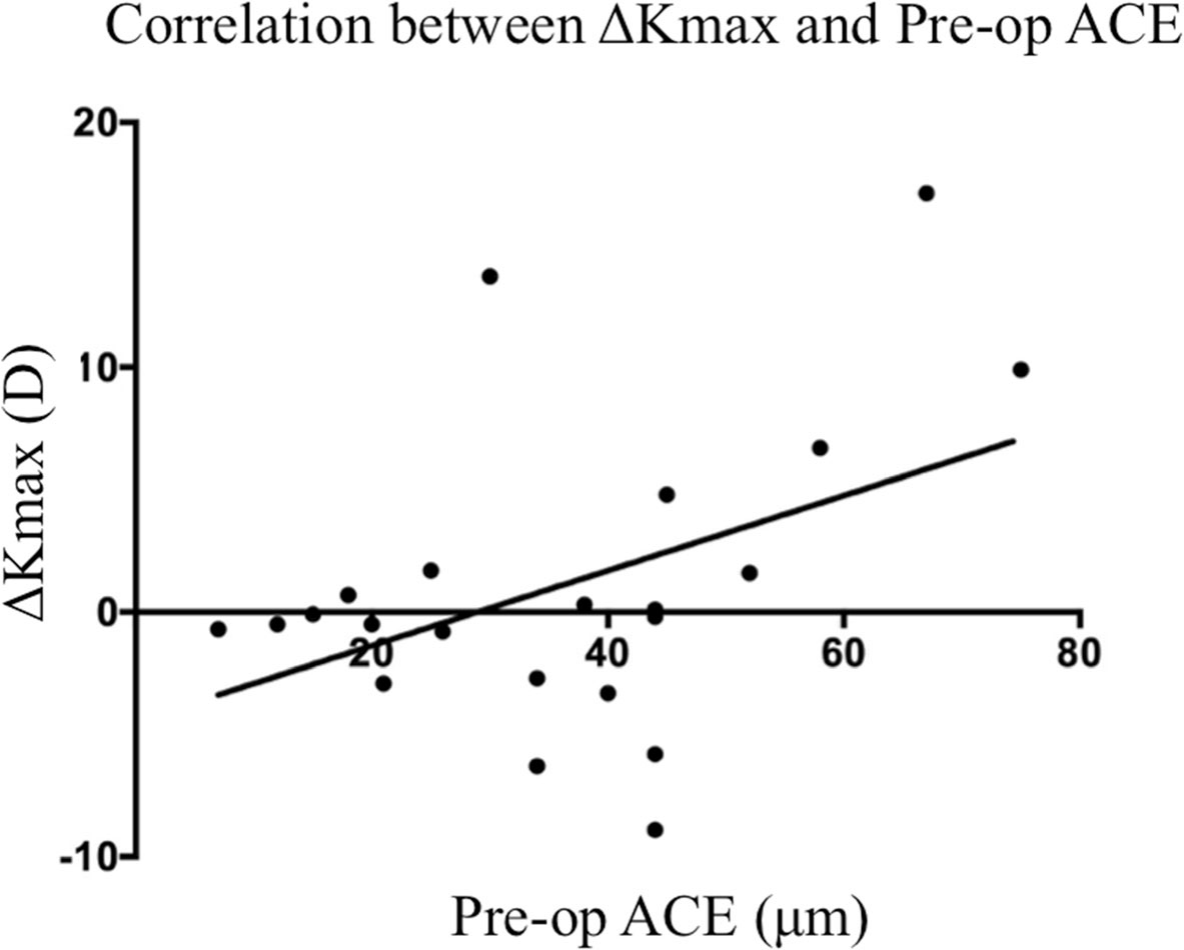
The correlation between ΔKmax and pre-operative ACE (*R* = 0.436, *P* = 0.043)

At post-operative month 24, corneal densitometry values of the anterior layer, central layer, posterior layer and total layer over Φ0-2 mm decreased by 4.09 ± 4.66, 2.47 ± 2.93, 2.00 ± 2.44 and 2.85 ± 3.15, respectively. Over Φ2–6 mm, those values decreased by 3.44 ± 2.60, 2.27 ± 1.61, 2.12 ± 1.56 and 2.12 ± 1.56, respectively. The increment of Kmax value (ΔKmax) was 1.09 ± 6.20D, however the decrements of ACE (ΔACE) and PCE (ΔPCE) were 4.4 ± 11.6 μm and 1.5 ± 14.1 μm, respectively. Pearson correlation test only revealed a significant correlation between ΔKmax and pre-operative ACE value (*R* = 0.436,*P* = 0.043), (Fig. 5). Further analysis showed that when all the patients were divided into two subgroups with the cut-off median preoperative Km of 50.30D (Subgroup 1 (*n* = 13): preoperative Km ≥ 50.30D; Subgroup 2 (*n* = 12): preoperative Km < 50.30D). Log-rank test showed that the Subgroup 1 progressed more significantly (7 of 13 cases progressed) when compared with the Subgroup 2 (none of 12 progressed), (F = 8.167, *P* = 0.004).

When these cases were divided into two subgroups with the cut-off median preoperative ACE of 35.3 μm (Subgroup 1 (n = 12): ACE ≥35.3 μm, Subgroup 2 (*n* = 13): ACE < 35.3 μm), Log-rank tests detected that the Subgroup 1 progressed (5 of 12 cases progressed) more remarkably when compared with the Subgroup 2 (2 of 13 progressed), (F = 5.207, *P* = 0.022).

## Discussion

A normal cornea is transparent and maintains a smooth and stable curvature [19]. Keratoconus compromises corneal biomechanical stability, leading to progressively increasing corneal keratometry and decreasing corneal thickness.

C-CXL is effective for retarding the progression of KC [20]. However, disadvantages to the standard therapy include the long procedure duration, the removal of the corneal epithelium, and the risks of infection and haze [12, 21, 22], which may lead to increased corneal densitometry and compromised transparency [11]. In the present study, we found the mean values of K1, K2, Km, Kmax, CA, CCT, TCT, ACE and PCE were unchanged over time, indicating that corneal structure remained stable during the 24-month follow-up. Moreover, as we expected [15], the pre-operative densitometry values of the full thickness of the cornea over annular regions Φ0-2 mm and Φ2–6 mm were higher than those of the control group (Figs. 3 and 4), but at post-operative month 24, these values remarkably decreased to normal levels. Strong evidence has shown that corneal collagen diameter increases and the gap between corneal collagen fasciculi narrows significantly after corneal crosslinking using riboflavin and UVA [8]. We hypothesize that the decreased corneal backscattering (densitometry) values are mainly due to the more tightly arranged corneal collagen fasciculi after ATE-CXL. In addition, we did not reveal any correlations between corneal densitometry values and ΔACE, or between the corneal densitometry values and ΔPCE. These results all imply that the densitometric variation might have little relationship with the changes in corneal shape.

So far, many protocols have been suggested for corneal cross-linking. The original cross-linking procedure, Dresden protocol (epithelium off, 3mw/cm^2^ UV-A irradiation for 30 min), demonstrated that both Kmax and Km readings kept stable, and ACE and PCE values continued to decrease up to 5 years after C-CXL [23]. Trans-epithelial corneal cross-linking (TE-CXL) procedure avoids epithelial removal. Magli A et al. [24] compared C-CXL and TE-CXL in progressive KC pediatric patients. They found TE-CXL provided similar efficacy and fewer complications than C-CXL at 1-year follow-up. Accelerated accelerated corneal cross-linking (A-CXL) protocols increase the power intensity of UV-A (9mw/cm^2^, 18mw/cm^2^, 30mw/cm^2^ and 45mw/cm^2^) and shorten the exposure time to achieve equal dosage. Meanwhile, A-CXL procedures could be performed with or without epithelium [15, 25]. A meta-analysis [26] concluded that, in general, ATE-CXL is effective and safe for KC stabilization, however, C-CXL results in greater flattening of corneal curvature. In the present study, we noticed that at post-operative month 24, 12 of 25 eyes (48%) had decreased Kmax values, 3 eyes (12%) had less than 1.00D shift in Kmax values, but the remaining 7 eyes had progressive KC (Kmax value increased more than 1.00D), implying that ATE-CXL may have insufficient efficacy in some specific KC patients. Interestingly, further analysis revealed that ΔKmax values were positively correlated with pre-operative ACE values (*R* = 0.436,*P* = 0.043), moreover, the Log-rank tests also showed that the subgroups with Km ≥ 50.30D or ACE ≥35.3 μm progressed significantly when compared with those with Km < 50.30D or ACE< 35.3 μm, indicating that severer KC patients (Amsler-Krumeich classification above Stage 2) may tend to have poorer outcomes (greater progression). Olivo-Payne A et al. [27] also reported that trans-epithelial accelerated corneal cross-linking demonstrated poorer long-term outcomes in severer cases, which was consistent to our findings.

CXL achieves its effect through increased formation of crosslinks in the corneal stroma, thus enhancing corneal biomechanical stiffness [28]. In severe KC, the cornea is generally thinner and less biomechanically stable [29] compared with mild KC. We hypothesize that, in advanced KC, there is greater stromal degeneration and fewer intact corneal collagen fibrils are available to be cross-linked [30] with ATE-CXL; hence, the effect of ATE-CXL on KC control in severe KC patients may be limited. Alternatively, more crosslinking formation (stronger treatment) may be required in severe cases to stabilize the biomechanically unstable cornea.

The current study had some limitations. First, we only assessed the 2-year changes in topographic parameters and densitometry values following ATE-CXL. The photopic, mesopic and scotopic visual acuity and higher order aberrations were not evaluated. It would be prudent to investigate the effects of ATE-CXL on visual quality in further studies. Second, the sample size is relatively small, however, we mainly investigate the changes in topographic and densitometric parameters before and after ATE-CXL, moreover, the KC patients and the myopes were matched for gender and age and thus, the selection bias should be limited.

## Conclusions

Topographic parameters, including K1, K2, Km, CA, Kmax, CCT, TCT, ACE, and PCE values, may all remain stable while the densitometry values of the total layer (over Φ0mm–2 mm and Φ2mm–6 mm) may decrease to normal levels at 2 years after ATE-CXL for KC. Patients with more severe KC may tend to have poorer outcomes. Further studies are essential to shed light on the optimal patient selection criteria for ATE-CXL.

## Abbreviations

ACE: Anterior corneal elevation
ATE-CXL: Accelerated transepithelial corneal cross-linking
CA: Corneal astigmatism
CCT: Central corneal thickness
C-CXL: Conventional corneal crosslinking
K1: The anterior simulated keratometer readings (Sim Ks) on a ring in 15° around the corneal apex along the flat meridian
K2: The anterior simulated keratometer readings (Sim Ks) on a ring in 15° around the corneal apex along the steep meridian
KC: Keratoconus
Km: The mean keratometry
Kmax: The steepest radius of the anterior corneal surface
PCE: Posterior corneal elevation
TCT: The thinnest corneal thickness
Φ: Annular diameter
